# Protocol for the OPTIMSE-1 randomised clinical trial to test specialist-led identification and management of cardio-renal-metabolic-pulmonary disease in machine learning algorithm-detected high-risk community-dwelling individuals

**DOI:** 10.1136/bmjopen-2025-101088

**Published:** 2025-08-06

**Authors:** Ramesh Nadarajah, Ali Wahab, Tobin Joseph, Catherine Reynolds, Sheena Bennett, Mohammad Haris, Adam B Smith, Chris Hayward, Jianhua Wu, Chris P Gale

**Affiliations:** 1 University of Leeds, Leeds, UK; 2 Department of Cardiology, Leeds Teaching Hospitals NHS Trust, Leeds, UK; 3 Leeds Institute of Data Analytics, Leeds, UK; 4 Leeds Institute of Cardiovascular and Metabolic Medicine, University of Leeds, Leeds, UK; 5 Queen Mary University of London, London, UK; 6 Leeds Teaching Hospitals NHS Trust Department of Cardiology, Leeds, UK

**Keywords:** Risk Factors, Electronic Health Records, Machine Learning, Clinical Trial, Cardiovascular Disease

## Abstract

**Introduction:**

People identified as higher risk by a machine learning algorithm (Future Innovations in Novel Detection of Atrial Fibrillation [FIND-AF]) are at increased risk of cardio-renal-metabolic-pulmonary disease and cardiovascular death. The OPTIMISE-1 randomised controlled trial aims to test the effect of community-based specialist-led identification and management of cardio-renal-metabolic-pulmonary (CRMP) disease and risk factors compared with usual care on the use of therapeutic interventions over a follow-up of 6 months among high FIND-AF risk community-dwelling individuals.

**Methods and analysis:**

OPTIMISE-1 is a multicentre, pragmatic, prospective, randomised, open-label, blinded-endpoint strategy trial that will recruit 138 participants aged 30 years or older, with a high FIND-AF risk score and previously enrolled in the FIND-AF pilot study (NCT05898165), to be randomised 1:1 to a specialist-led care intervention or usual care. The primary endpoint is a composite of initiation or increase of guideline-directed CRMP therapies. The secondary endpoints are the components of the primary endpoint, time to primary endpoint, diagnosis of new CRMP diseases or risk factors, time to diagnosis of new CRMP diseases or risk factors, initiation or increase of guideline-directed CRMP therapies for participants with recorded CRMP disease, initiation or increase of guideline-directed CRMP therapies for participants with newly diagnosed CRMP disease and change in participant-reported quality of life.

**Ethics and dissemination:**

The study has ethical approval (the North East & North Tyneside 2 Research Ethics Committee reference 24/NE/0188). Findings will be announced at relevant conferences and published in peer-reviewed journals in line with the Funder’s open access policy.

**Trial registration number:**

Clinicaltrials.gov NCT06444711.

**STRENGTHS AND LIMITATIONS OF THIS STUDY**

- Future Innovations in Novel Detection of Atrial Fibrillation is a pragmatic, scalable machine learning algorithm derived from routine electronic healthcare data, which facilitates translation to other clinical contexts.
- The trial aims to improve treatment across a range of cardio-renal-metabolic-pulmonary diseases which may enhance the therapeutic benefit achieved for participants.
- Though the trial will establish if more care for cardio-renal-metabolic-pulmonary risk factors can be provided through specialist-led services in the community for high-risk participants, it will not determine if this results in improved clinical outcomes.
- Whether a specialist providing this pathway is cost-effective will require further assessment.

## Introduction

 Treatment of hypertension, dyslipidaemia, diabetes mellitus, chronic kidney disease (CKD) and obesity has been shown in randomised clinical trials (RCT) to reduce the risk of cardiovascular disease and cardiovascular death.^1^,^6^ Many risk factors for cardiovascular disease do not occur in isolation, but often coexist in clusters for an individual (as a syndemic) and increase the risk of the development of other cardiovascular risk factors.^1^ Research has demonstrated that, especially during the COVID-19 pandemic, the management of cardio-renal-metabolic-pulmonary (CRMP) risk factors through monitoring of observations, blood tests and prescription of medications has deteriorated.^7^ Thus, patients may not have CRMP diseases detected, recorded and/or treated in routine primary care.

The Future Innovations in Novel Detection of Atrial Fibrillation (FIND-AF) risk score (a UKCA Class I medical device) is a fixed machine learning (ML) algorithm that uses data in primary care electronic health records (EHRs) to identify people at higher risk of receiving a diagnosis of atrial fibrillation (AF) in the next 6 months. In follow-on analyses, we demonstrated that individuals identified as higher FIND-AF risk are also at increased risk of other incident CRMP conditions including CKD, heart failure, diabetes mellitus, stroke/transient ischaemic attack, myocardial infarction, chronic obstructive pulmonary disease (COPD) and peripheral vascular disease.^8^ We have also shown that higher FIND-AF predicted risk individuals, compared with lower FIND-AF predicted risk individuals, are at a more than 10-fold higher risk of death—the majority of which are of cardiovascular cause—over a 10-year follow-up period.^8^ Accordingly, individuals at higher FIND-AF predicted risk may have comorbidities and cardiovascular risk factors that are undiagnosed or undertreated during routine practice that may be potential targets for guideline-directed treatment to reduce the risk of future adverse events.

The OPTIMISE-1 RCT aims to test the effect of community-based specialist-led identification and management of CRMP disease and risk factors compared with usual care on use of therapeutic interventions over a follow-up of 6 months among high FIND-AF risk community-dwelling individuals.

## Methods and analysis

### Study design

**Figure 1:**
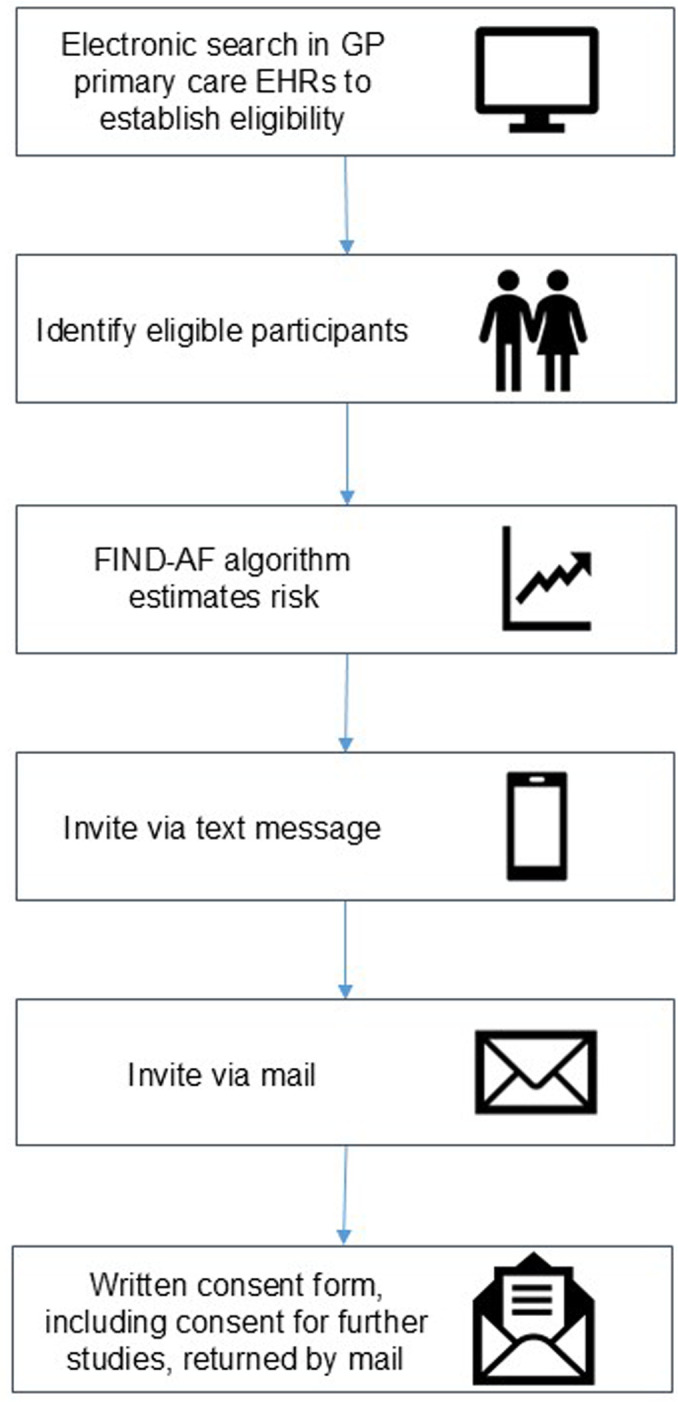
Participant recruitment from the FIND-AF study. FIND-AF, Future Innovations in Novel Detection of Atrial Fibrillation.

This publication describes V1.0 of the OPTIMISE-1 study protocol, dated 23 September 2024. The OPTIMISE-1 study is an interventional, multicentre, pragmatic, prospective, randomised, open-label, blinded-endpoint trial (figure 1). This protocol is reported in accordance with the Standard Protocol Items: Recommendations for Interventional Trials statement.^9^

### FIND-AF score

Derivation and validation of the FIND-AF score have been described elsewhere.^10^ Briefly, it is a random forest classifier trained to predict AF within a 6-month prediction horizon. Random forest is a machine learning method consisting of many individual decision trees that operate as an ensemble. FIND-AF was trained using 10-fold cross validation on the training dataset. Model performance for FIND-AF was determined using a holdout test dataset with internal bootstrap validation with 200 samples. The area under the receiver operating characteristic curve for incident AF was 0.824 (95% CI 0.814 to 0.834).^10^

The variables included in FIND-AF were restricted to age, sex, comorbidities and ethnicity to enhance scalability and international generalisability.^10^ The predictor variables were selected from a prior systematic review.^11^ Ethnicity information is routinely collected in the UK NHS, and FIND-AF also included an ‘ethnicity unrecorded’ category where it was unavailable because missingness was considered to be informative.^10^ Thus, none of the variables are expected to be missing in routine data, and the algorithm could be applied at scale across a dataset of over 2 million UK primary care EHRs in the original derivation paper.^10^ The performance of FIND-AF for the prediction of incident AF was found to be equally robust in both men and women and across ethnic groups.^10^ The importance of variables for the calculation of the FIND-AF score is demonstrated by the Gini impurity measure.^10^

### FIND-AF for incident cardio-renal-metabolic-pulmonary diseases

In a follow-on analysis using routinely collected UK primary care EHRs, we found higher FIND-AF risk, compared with lower FIND-AF risk, was associated with incident chronic obstructive pulmonary disease (cumulative incidence per 1000 persons at 10 years 395.8; HR 2.02, 95% CI 2.00 to 2.05; median time to event 2.68 years), chronic kidney disease (245.2; 6.85, 6.70–7.00; 5.44), heart failure (124.7; 12.54, 12.08–13.01; 4.06), diabetes mellitus (123.3; 2.05, 2.00–2.10; 3.45), stroke/transient ischaemic attack (118.9; 8.07, 7.80–8.34; 4.27), myocardial infarction (69.6; 5.02, 4.82–5.22; 4.32), peripheral vascular disease (44.6; 6.62, 6.28–6.98; 4.28), valvular heart disease (37.8; 6.49, 6.14–6.85; 4.54), aortic stenosis (18.7; 9.98, 9.16–10.87; 4.41), and death from any cause (273.9; 10.45, 10.23–10.68; 4.75).^8^ The higher risk group constituted 74% of deaths from cardiovascular or cerebrovascular causes (8582/11 676). On subgroup analysis, higher FIND-AF risk, compared with lower FIND-AF risk, was associated with increased incidence for each of the outcomes in both men and women and in younger (age 30–64 years) and older (age ≥65 years) patients.

### FIND-AF pilot

In the FIND-AF pilot study, 1960 patients with a primary care EHR aged 30 years and over, without a preceding diagnosis of AF or atrial flutter (AFl) and had a CHA_2_DS_2_-VASc score ≥2 or women with a CHA_2_DS_2_-VASc score ≥3, were enrolled.^12^ Individuals receiving any form of anticoagulation and those on the palliative care register were excluded. Each consenting participant had their FIND-AF score calculated from their primary care EHR. A FIND-AF score of 0.0053 (equating to the top decile of risk in the general population) was used as the threshold to define high risk.^12^

### Study population

**Figure 2:**
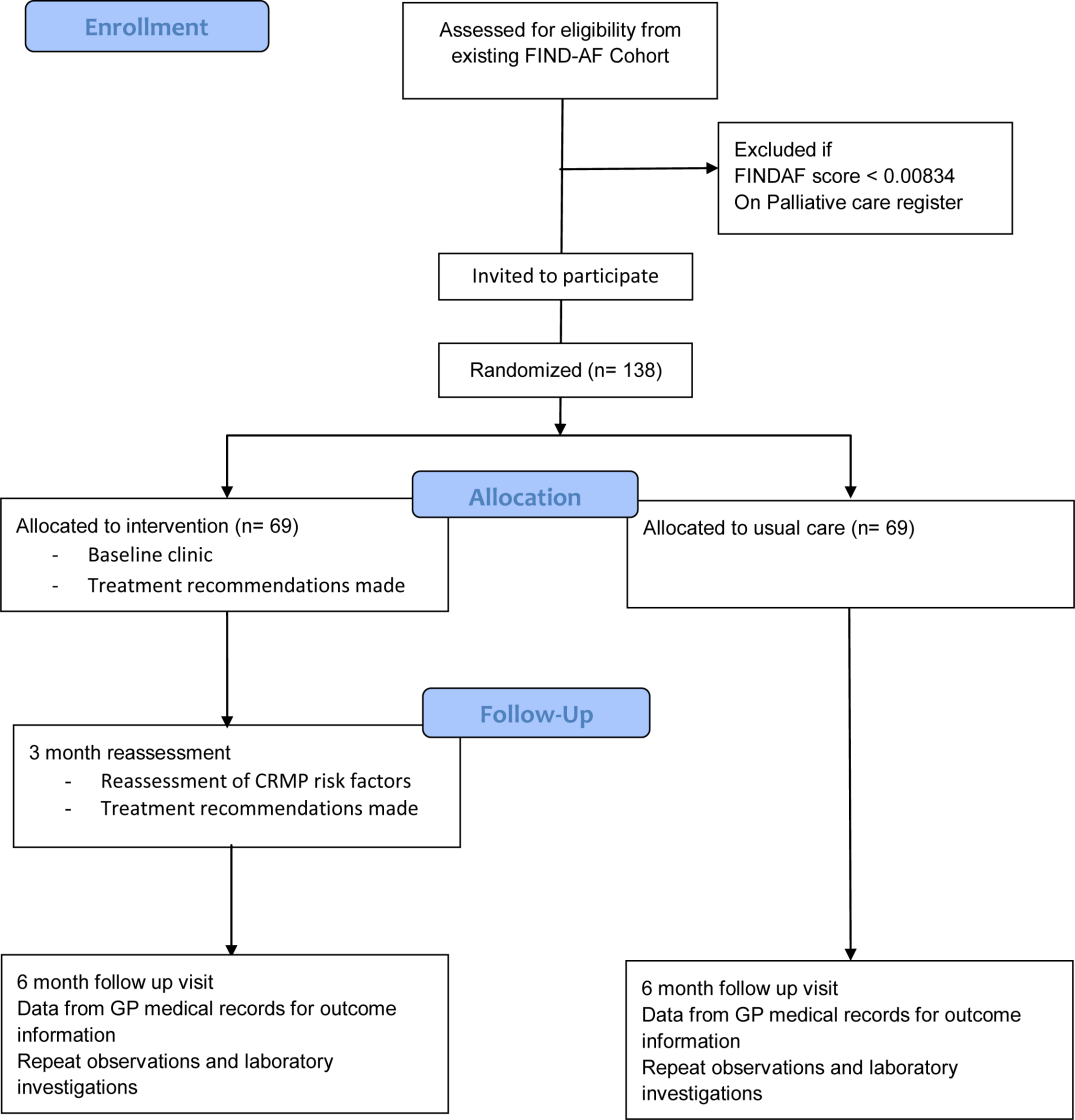
CONSORT flow diagram for proposed participant flow through the trial.

The study will enrol 138 participants aged 30 years and over with a primary care EHR at General Practices in the NIHR Research Delivery Network Yorkshire and Humber region who were recruited into the FIND-AF pilot study (NCT05898165) and consented to be contacted about further studies, thereby forming the FIND-AF cohort-RCT platform (figure 2).^12 13^

The inclusion and exclusion criteria for OPTIMISE-1 are summarised in box 1. Participants diagnosed with AF or AFl during the FIND-AF pilot study, or as part of routine care, are eligible for OPTIMISE-1. For OPTIMISE-1, for eligibility for inclusion, a FIND-AF score >0.00834, which equates to the top 5% of risk in the general population is required; ~25% of the participants in the FIND-AF study were in this risk category.^12^

Box 1 **Inclusion and exclusion criteria for the OPTIMISE-1 randomised clinical trial**

**Inclusion criteria**

- Participants in the FIND-AF study who have consented to be contacted about participation in further studies
- FIND-AF score ≥0.00834

**Exclusion criteria**

- Unable to give written informed consent for participation in the study
- Unable to adhere to the study requirements

### Enrolment

Eligible participants in the FIND-AF pilot study will be identified and checked to ensure they meet the OPTIMISE-1 RCT study inclusion and exclusion criteria (including that they still have the capacity to provide consent). Eligible individuals from the study team will be invited by letter and/or telephone to participate. All participants will be required to provide written informed consent by returning a completed consent form to the research team. In the same postal invite, a health-related quality of life questionnaire (EQ-5D-5L) will be provided for all consenting participants.

### Randomisation

Following completion of consent and the questionnaire, each participant will be randomised using three strata (age, sex, FIND-AF risk score) to ensure balance for key baseline variables. Eligible participants will be randomised in a 1:1 ratio to the intervention arm or usual care. Given that the trial is open label, participants will not be blinded to whether they are in the intervention arm or control arm. For participants in the control arm, their primary care EHRs will be reviewed for relevant CRMP risk factors and diseases, and they will be followed up in person at a research visit in the community at 6 months follow-up.

### Intervention

#### Baseline clinic attendance

Participants randomly allocated to the intervention will be invited to attend a community-based research visit with a research team medical specialist (cardiology) and will receive blood pressure, heart rate, height and weight measurements, auscultation of heart and lungs, undergo spirometry and the taking of a clinical history. Their records will be reviewed for relevant CRMP blood tests and if they have not been taken but routinely would be recommended by the United Kingdom National Institute for Health and Care Excellence (NICE) guidance, they will be taken. These blood tests include estimated glomerular filtration rate, glycosylated haemoglobin (HbA1c) and a lipid profile. A urinary albumin to creatinine ratio will also be measured if required, and no recent result is available.

#### Intervention arm study procedures

Based on the results from their baseline assessment, the research team specialist will recommend investigations and treatments for intervention arm participants that are recommended by NICE guidelines. These recommendations will be informed to the participant and the participant’s General Practitioner (GP); a results letter will be provided to the participant’s general practitioner. Participating GP practices will add diagnoses, investigations and treatments to the participant’s primary care electronic health records.

The participants in the intervention arm will then attend a second research visit in the community at 3 months following randomisation. At this appointment, the participant will be reviewed by the research team specialist, and based on the available data, further changes to treatment to adhere to NICE guidelines recommended.

#### Follow-up assessments

Participants in both the control and intervention arms will attend an appointment in the community at 6 months after randomisation. Data pertinent to the study endpoints will be collected from the participant’s primary care EHR. Further observations and laboratory investigations will be taken to allow ascertainment of study outcomes, and the participant will complete an EQ-5D-5L questionnaire.

The intervention will be for 6 months from randomisation, with follow-up through EHRs at 5 years and 10 years after enrolment into the FIND AF pilot study. A results letter from the follow-up assessment will be provided to participants and their General Practitioners. Ongoing care of patients after the end of the 6-month follow-up will be at the discretion of the participant and their general practitioner.

### Baseline characteristics

Baseline demographics, comorbidities and medications of participants that are continuous variables will be reported as mean ± SD deviation and categorical variables reported as frequencies with corresponding percentages. Baseline characteristics are demonstrated in box 2.

Box 2 **Participant baseline characteristics**

**Participant characteristics**

- Age
- Sex
- Ethnicity

**Medical history**

- Coronary artery disease
- Chronic kidney disease
- Peripheral vascular disease
- Heart failure
- Hypertension
- Diabetes mellitus
- Stroke/transient ischaemic attack
- Valvular heart disease
- Chronic obstructive pulmonary disease
- Current smoking status

**Medications**

- Aspirin
- Angiotensin-converting enzyme (ACE) inhibitor or angiotensin receptor blocker
- Beta blocker
- Calcium channel blocker
- Thiazide-like diuretic
- Spironolactone
- Sodium-glucose co-transporter 2 inhibitor
- Glucagon-like peptide-1 receptor agonist/glucagon-like peptide-1 receptor agonist and gastric inhibitory peptide agonist
- HMG Co-A reductase inhibitor (statin)
- Ezetimibe
- Proprotein convertase subtilisin/kexin type 9 inhibitor
- Metformin
- Dipeptidyl peptidase-4 inhibitor
- Sulphonylurea
- Insulin
- Orlistat
- Nicotine replacement therapy
- Bupropion
- Inhaler therapy

### Outcomes

#### Primary outcome

The primary outcome is the proportion of patients with initiation and/or increase in the dose of any of the following therapeutic interventions to optimise CRMP disease and/or risk factor management including:

- Renin-angiotensin system inhibitors with multiple indications: ACE inhibitor/angiotensin 2 receptor blocker;
- Hypertension management: calcium channel blocker/thiazide-like diuretic/spironolactone/alpha blocker/beta blocker;
- Glycaemic agents with cardiovascular benefit: sodium glucose co-transporter 2 (SGLT2) inhibitor, glucagon-like peptide-1 receptor agonist (GLP-1 RA);
- Glycaemic agents without cardiovascular benefit: metformin/dipeptidyl peptidase 4 (DPP4) inhibitor/pioglitazone/sulphonylurea/insulin;
- Lipid modification agents: statin/ezetimibe/icosapent ethyl/bempedoic acid/proprotein convertase subtilisin/kexin type 9 (PCSK9) inhibitor;
- Obesity-specific treatment: orlistat/GLP-1 RA (liraglutide, semaglutide) or GLP-1 RA/gastric inhibitory peptide agonist (tirzepatide);
- Smoking cessation: nicotine replacement therapy/bupropion; and
- Respiratory inhaler therapy: short-acting beta agonist/short-acting muscarinic antagonist/long-acting beta agonist/long-acting muscarinic antagonist/inhaled corticosteroid—or combination inhalers.

#### Secondary outcome

The secondary outcomes are as follows:

- Components of primary endpoint;
- ime to primary endpoint;
- Diagnosis of new CRMP risk factor (hypertension, diabetes, CKD, obesity, current smoking, COPD);
- time to diagnosis of a new CRMP risk factor;
- Uptake of guideline-directed risk factor management in those with recorded CRMP risk factors;
- Uptake of guideline-directed risk factor management in those with newly diagnosed CRMP risk factors;
- Change in EQ-5D-5L questionnaire score between randomisation and 6-month follow-up.

### Sample size

Feasibility work in 30 high FIND-AF risk patients demonstrated that of patients at high FIND-AF risk in routine care, for cardio-renal-metabolic-pulmonary risk factors, there is undertreatment.^14^ Of high FIND-AF risk patients with hypertension, 58.5% (31/53) of those aged <80 years had a systolic blood pressure (SBP) >140 mm Hg, and 54.5% (6/11) of those aged ≥80 years had an SBP>150 mm Hg. Of those with type 2 diabetes and coexistent CVD, only 23.1% (3/13) were on SGLT2 inhibitor therapy. Of higher risk patients on statin therapy, 37.0% (20/54) had LDL-cholesterol >1.8 mmol/L, and 23.1% (3/13) of patients with previous CVD had an LDL-cholesterol >2.0 mmol/L. Furthermore, 19.5% (16/82) of the higher-risk cohort had undiagnosed moderate or high-risk CKD. Those with unrecognised CKD were often not on a statin (41.7%; 5/12), ACE-i/ARB therapy with coexistent hypertension (61.5%; 8/13), or an SGLT2 inhibitor with coexistent diabetes (50.0% (3/6), 83.3% (5/6), respectively). Almost half of the cohort (49%) were found to be obese but not previously recorded, and 17% (14/82) were eligible for GLP-1 RA therapy.^15^

Thus, we conservatively estimate that 40% of the intervention group will meet the primary outcome, with 10% meeting the primary outcome in the control group. We estimate that there will be a 5% dropout. Even if the primary outcome was only met in 30% of the intervention group (75% of the expected effect size), a sample size of 138 participants randomised 1:1 to intervention and control would give a 90% power to detect a difference with α = 0.05. Furthermore, this sample size will enable the balancing of important baseline variables defined at three strata (FIND-AF risk score, age and sex), allowing for two levels for each stratum (absolute FIND-AF risk scores within the high-risk cohort, age dichotomised above and below 75 years, men and women) and at least 15 patients in each level of each stratum.

### Statistical analysis

#### Primary outcome

The study population for the primary analysis will be the intention-to-treat analysis among all randomised patients irrespective of compliance. The Odds Ratio (OR) of the primary outcome between the intervention arm and the control arm will be calculated.

#### Secondary outcomes

The OR of each component of the primary outcome will be calculated. ORs will also be calculated for the new diagnosis of CRMP risk factors.

For time to the primary outcome and time to new diagnosis of a CRMP risk factor will be plotted as cumulative incidence over the study period, and hazard ratios (potentially adjusted) will be calculated to understand differences between the intervention and control arms.

For the secondary outcomes relating to guideline adherence for each risk factor (online supplemental table 1), the study population will be subgrouped by the presence of that risk factor at baseline. An all-or-none scoring composite performance measure will be used as an aggregation method to define receipt of optimal care, that is, patients who received all of the care interventions for which they are eligible will be considered to have received optimal care, but patients missing one or more interventions will be considered to have received suboptimal care. If the data are missing, the patient will be assumed not to have received the intervention. Overall, receipt of optimal care will be split into four categories: no interventions received, <40% of eligible interventions received, ≥40% to <80% of eligible interventions received and ≥80% of interventions received. Categorical data will be described using numbers and percentages for non-missing data, and continuous data were described using medians, IQR, means and SD. Comparison between intervention and control arms will be performed using the Kruskal–Wallis test or using Pearson’s χ2 test or Fisher’s exact test if any count is <5. Cumulative adherence, counting the percentage of patients in whom at least one guideline recommendation is applied, will be plotted.

For the quality of life, the difference in EQ-5D-5L domains and visual analogue scores at baseline and at 6-month follow-up will be calculated and compared between the intervention arm and control arm using an independent sample t-test.

### Patient and public involvement

The FIND-AF patient and public involvement group has informed the FIND-AF research programme from inception. They have confirmed that they believe the proactive management of cardiovascular, renal, metabolic and pulmonary disease and risk factors is an important priority to improve the health and well-being of the population. Furthermore, they have highlighted that the investigation of technology that uses routinely collected data to provide a more personalised approach to care is worthwhile.

### Limitations

There are a number of potential limitations to this study. First, the sample is derived from the National Health Service (NHS) in one region of the United Kingdom; therefore, the results might not be generalisable. However, we have attempted to control for this by selecting general practices in areas with high ethnic diversity, wide socio-economic ranges and a population that has premature cardiovascular morbidity and mortality. Second, 6 months of follow-up from the time of randomisation may be too short a duration to identify significant differences in the prespecified outcomes according to the intervention. Even if this is so, participants of OPTIMSE-1 form part of the FIND-AF longitudinal study and will have follow-up for several years in a prespecified cohort-RCT design.^13^ This protocol is intended for participants that undergo specific screening for CRMP risk factors. Thirdly, the FIND-AF algorithm was not developed for this purpose and was intended to screen for AF. However, the algorithm has already been externally validated in much larger cohorts and has been able to identify those that are at higher risk of developing several of the conditions we are interested in, and therefore, it is worth investigating prospectively. Fourth, for translation to clinical practice, the optimum approach to providing the FIND-AF risk stratification to primary care teams needs to be defined. Formation of a FIND-AF digital health platform that can integrate with primary care EHRs and provide contemporaneous estimates of FIND-AF risk at the patient level is the subject of funding applications. A qualitative study has already demonstrated that primary care healthcare professionals are enthusiastic to be able to use risk stratification through FIND-AF and recommended the formation of high FIND-AF risk lists at the practice level as a medium through which the practice could organise clinical activities.^16^ Fifth, model performance and association of model score with outcomes may change over time as the prevalence of predictors and association of predictors with the outcomes change over time. As discussed in the original protocol for the development and validation of FIND-AF,^17^ the model itself could be reviewed and updated by a prespecified expert consensus group on an annual basis after incorporating evidence from post-service utilisation and the curation of more data.

### Ethics and dissemination

The study will be performed in compliance with the articles of the Declaration of Helsinki (revised in October 2013). The study has ethical approval (the North East & North Tyneside 2 Research Ethics Committee reference 24/NE/0188), and the study was approved by the Health Research Authority (IRAS project ID: 343919) and registered on Clinical Trials.gov (NCT06444711). Study results will be disseminated at relevant conferences and published in peer-reviewed journals. Authorship will be decided according to ICMJE guidelines as to qualifying contributions, and the primary results manuscript jointly drafted by the co-chief investigators and the trial methodologists before circulating to remaining co-authors. Following the analysis, participants will be invited to a webinar where the research team will present the results of the study and provide participants with an opportunity to ask questions.

## Discussion

Cardiovascular disease causes a quarter of all deaths in the UK and is the largest cause of premature mortality in deprived areas.^18^ The NHS of England Long Term Plan emphasises that this is the single biggest area where the NHS can save lives and that better detection and treatment of cardiovascular, renal, metabolic and pulmonary risk factors is a priority.^19^ The European Society of Cardiology’s (ESC) Digital Cardiology and Artificial Intelligence Committee aims to advance the care for cardiovascular health through innovative technologies that enhance physicians’ ability to prevent, diagnose, and manage heart disease. The EUROASPIRE V survey highlighted large proportions of people at higher risk for cardiovascular disease have inadequate control of other cardiovascular risk factors such as diabetes, dyslipidaemia and hypertension. They concluded that ‘the potential to reduce the risk of future cardiovascular disease throughout Europe by improved preventive cardiology programmes is substantial’. Thus, we aim to address these treatment gaps in this ML-directed study.^20^

The OPTIMISE-1 RCT will provide novel data for a specialist-led intervention in primary care in a high-risk cohort identified by an ML algorithm to improve the treatment of CRMP diseases and risk factors. If the treatment of high-risk patients can be improved, then a follow-on RCT will explore if this can reduce healthcare utilisation, cardiovascular hospitalisation and cardiovascular death.

## Supplementary material

10.1136/bmjopen-2025-101088 online supplemental file 1

## References

[1] Williams B, Mancia G, Spiering W (2018). 2018 ESC/ESH Guidelines for the management of arterial hypertension: The Task Force for the management of arterial hypertension of the European Society of Cardiology (ESC) and the European Society of Hypertension (ESH). Eur Heart J.

[2] Marx N, Federici M, Schütt K (2023). 2023 ESC Guidelines for the management of cardiovascular disease in patients with diabetes: Developed by the task force on the management of cardiovascular disease in patients with diabetes of the European Society of Cardiology (ESC). Eur Heart J.

[3] Visseren FL, Mach F, Smulders YM (2021). 2021 ESC Guidelines on cardiovascular disease prevention in clinical practice: Developed by the Task Force for cardiovascular disease prevention in clinical practice with representatives of the European Society of Cardiology and 12 medical societies With the special contribution of the European Association of Preventive Cardiology (EAPC). Eur J Prev Cardiol.

[4] Ortiz A, Wanner C, Gansevoort R (2022). Chronic kidney disease as cardiovascular risk factor in routine clinical practice: a position statement by the Council of the European Renal Association. Eur J Prev Cardiol.

[5] Zelniker TA, Wiviott SD, Raz I (2019). SGLT2 inhibitors for primary and secondary prevention of cardiovascular and renal outcomes in type 2 diabetes: a systematic review and meta-analysis of cardiovascular outcome trials. Lancet.

[6] Yumuk V, Tsigos C, Fried M (2015). European Guidelines for Obesity Management in Adults. Obes Facts.

[7] Dale CE, Takhar R, Carragher R (2023). The impact of the COVID-19 pandemic on cardiovascular disease prevention and management. Nat Med.

[8] Wu J, Nadarajah R, Nakao YM (2023). Incident cardiovascular, renal, metabolic diseases and death in individuals identified for risk-guided atrial fibrillation screening: a nationwide cohort study. Open Heart.

[9] Chan A-W, Tetzlaff JM, Gøtzsche PC (2013). SPIRIT 2013 explanation and elaboration: guidance for protocols of clinical trials. BMJ.

[10] Nadarajah R, Wu J, Hogg D (2023). Prediction of short-term atrial fibrillation risk using primary care electronic health records. Heart.

[11] Nadarajah R, Alsaeed E, Hurdus B (2022). Prediction of incident atrial fibrillation in community-based electronic health records: a systematic review with meta-analysis. Heart.

[12] Nadarajah R, Wahab A, Reynolds C (2023). Future Innovations in Novel Detection for Atrial Fibrillation (FIND-AF): pilot study of an electronic health record machine learning algorithm-guided intervention to identify undiagnosed atrial fibrillation. Open Heart.

[13] Wahab A, Nadarajah R, Reynolds C (2024). Phenotypic characterization of people at risk of atrial fibrillation: protocol for the FIND-AF longitudinal cohort study. Eur J Prev Cardiol.

[14] Nadarajah R, Wahab A, Reynolds C (2024). Development, validation and prospective clinical implementation of a machine learning algorithm for incident cardio-renal-metabolic diseases and cardiovascular death: the OPTIMISE study. Eur J Prev Cardiol.

[15] Nadarajah R, Wahab A, Reynolds C (2024). 204 Machine learning for incident cardio-renal-metabolic disease and cardiovascular death: the optimise study. Heart.

[16] Hamilton E, Shone L, Reynolds C (2025). Perceptions of healthcare professionals on the use of a risk prediction model to inform atrial fibrillation screening: qualitative interview study in English primary care. BMJ Open.

[17] Nadarajah R, Wu J, Arbel R (2023). Risk of atrial fibrillation and association with other diseases: protocol of the derivation and international external validation of a prediction model using nationwide population-based electronic health records. BMJ Open.

[18] British Heart Foundation (2024). UK factsheet. https://www.bhf.org.uk/-/media/files/for-professionals/research/heart-statistics/bhf-cvd-statistics-uk-factsheet.pdf?rev=5c76af77f68e4c43b19f957890005bbe&hash=D31DB43089AAD361320212D15D4B70FB.

[19] NHS (2019). Cardiovascular disease. https://www.longtermplan.nhs.uk/areas-of-work/cardiovascular-disease.

[20] Kotseva K, De Backer G, De Bacquer D (2021). Primary prevention efforts are poorly developed in people at high cardiovascular risk: A report from the European Society of Cardiology EURObservational Research Programme EUROASPIRE V survey in 16 European countries. Eur J Prev Cardiol.

