# Supplementary material for: Protocol for the OPTIMSE-1 randomised clinical trial to test specialist-led identification and management of cardio-renal-metabolic-pulmonary disease in machine learning algorithm-detected high-risk community-dwelling individuals
Source: BMJ Open. 2025 Aug 6;15(8):e101088. doi: 10.1136/bmjopen-2025-101088 (PMC12336468; doi:10.1136/bmjopen-2025-101088)
Supplement: online supplemental file 1 [file bmjopen-15-8-s001.docx]

**Supplementary Table 1.** National Institute of Health and Care Excellence guideline adherence in those with recorded cardio-renal-metabolic-pulmonary risk factors:

| Condition | Target |
| --- | --- |
| Hypertension | Clinic BP <150/90 mmHg when aged ≥80 years  Clinic BP <140/90 mmHg when aged <80 years  Clinic BP <130/80 mmHg if chronic kidney disease with albumin:creatinine ratio ≥70mg/mmol |
| Dyslipidaemia | Primary prevention (without known vascular disease but estimated 10‑year risk of CVD is ≥10%): Non-HDL-C level reduced by >40%  Secondary prevention (with known vascular disease): LDL-Cholesterol ≤2.0 mmol/l or non-HDL-Cholesterol ≤2.6 mmol/l |
| Diabetes mellitus | HbA1c ≤48 mmol/mol (6.5%) if on a single drug not associated with hypoglycaemia or managed by lifestyle or if type I diabetic  HbA1c ≤53 mmol/mol (7.0%) if on a drug associated with hypoglycaemia  Treatment with a SGLT2 inhibitor if co-existent heart failure, cardiovascular disease, or chronic kidney disease |
| Chronic kidney disease | Treatment with a statin  Treatment with ACEi/ARB if hypertensive or diabetic + albumin:creatinine ratio ≥3mg/mmol  Treatment with SGLT2i if on ACEi/ARB and eGFR 25-75ml/min/1.73m^2^ and if type 2 diabetes or albumin:creatinine ratio ≥ 22.6mg/mmol  Antiplatelet for secondary prevention of cardiovascular disease |
| Obesity | Behavioural intervention  Pharmacological treatment   - Orlistat if BMI ≥30 kg/m^2^ or >28 kg/m^2^ with associated risk factors (dyslipidaemia, hypertension, diabetes mellitus) - Liraglutide if BMI ≥35 kg/m^2^ or at least 32.5 kg/m^2^ for members of minority ethnic groups known to be at equivalent risk of the consequences of obesity at a lower BMI and they have non-diabetic hyperglycaemia and high risk of cardiovascular disease and prescribed in tier 3 weight management service - Semaglutide if BMI ≥35 kg/m^2^ or BMI 30-35 kg/m^2^ and meet criteria for referral to specialist weight management services (considering liraglutide, consideration of surgery, conventional treatment has failed, complex disease needs, underlying aetiology needs assessment) - Tirzepatide [as adjunct to reduced calorie diet and increased physical activity] if BMI ≥ 30 kg/m^2^ or 27-30 kg/m^2^ in the presence of at least one weight-related comorbid condition such as hypertension, dyslipidaemia, obstructive sleep apnoea, cardiovascular disease, prediabetes, or type 2 diabetes mellitus.   Bariatric surgery   - Adults with BMI ≥35 kg/m^2^ who have been diagnosed with type 2 diabetes within the past 10 years are offered a referral for bariatric surgery assessment - Adults with a BMI >50 kg/m2 are offered a referral for bariatric surgery assessment   Referral to weight management services   - The underlying causes of overweight and obesity need to be assessed. - The person has complex disease states or needs that cannot be managed adequately in tier 2 (for example, the additional support needs of people with learning disabilities). - Conventional treatment has failed in primary or secondary care. - Drug treatment is being considered for a person with a BMI of more than 50 kg/m2. - Specialist interventions (such as a very-low-calorie diet) may be needed. - Surgery is being considered. - Treatment with liraglutide (Saxenda®) is being considered. |
| Smoking cessation | Referral to smoking cessation services  Offer of nicotine replacement therapy or buproprion |
| COPD | Referral to pulmonary rehabilitation  Advice as per smoking cessation  Referral to dietician if BMI is abnormal  Escalation of inhaler therapy if required   - Bronchodilator if modified Medical Research Council (mMRC) dyspnoea score 0-1 and COPD assessment test (CAT) <10 and 0-1 exacerbation not leading to hospitalisation - Long acting beta agonist (LABA)+ Long acting muscuarinic agonist (LAMA) if mMRC ≥2 and CAT ≥10 and 0-1 exacerbation not leading to hospitalisation - LABA + LAMA if 2 or more moderate exacerbations or ≥1 hospitalisation due to exacerbation. Can consider addition of inhaled corticosteroid (ICS) if eosinophils ≥300 cells/microlitre   Consider change of delivery system and re-assessment of inhaler technique before any change in treatment  Referral to specialist care   - If lung cancer suspected - Uncertainty of diagnosis - Very severe or rapidly worsening - Cor pulmonale is suspect - Age <40 or family history of alpha-1-antitrypsin deficiency - Frequent infections - Consideration of oxygen therapy - Consideration of long term non invasive ventilation - Consideration of nebulizer therapy or long term oral corticosteroids - Consideration of lung surgery |
