# Supplementary material for: Protocol for the OPTIMSE-1 randomised clinical trial to test specialist-led identification and management of cardio-renal-metabolic-pulmonary disease in machine learning algorithm-detected high-risk community-dwelling individuals
Source: BMJ Open. 2025 Aug 6;15(8):e101088. doi: 10.1136/bmjopen-2025-101088 (PMC12336468; doi:10.1136/bmjopen-2025-101088)
Supplement: Supplementary file 2 [file bmjopen-2025-101088.reviewer_comments.pdf]

PEER REVIEW HISTORY

BMJ Open publishes all reviews undertaken for accepted manuscripts. Reviewers are asked to complete a checklist review form and are provided with free text boxes to elaborate on their assessment. These free text comments are reproduced below.

ARTICLE DETAILS

Title (Provisional)

Protocol for the OPTIMSE-1 randomised clinical trial to test specialist-led identification and management of cardio-renal-metabolic-pulmonary disease in machine learning algorithm-detected high risk community-dwelling individuals.

Authors

Nadarajah, Ramesh; Wahab, Ali; Joseph, Tobin; Reynolds, Catherine; Bennet, Sheena; Harris, Mohammad; Smith, Adam B; Hayward, Chris; Wu, Jianhua; Gale, Chris

---

VERSION 1 - REVIEW

**Reviewer**	**1**
**Name**	**Ibrahim, Mahmoud Ashraf**
**Affiliation**	**EDC , Center for Diabetes Education, Diabetes**
**Date**	**15-Apr-2025**
**COI**	**None**

April 15th 2025

Reviewer's report:

Thank you very much for giving me the chance to review the paper entitled:

“Protocol for the OPTIMSE-1 randomised clinical trial to test specialist-led identification and management of cardiorenal-metabolic-pulmonary disease in machine learning algorithm-etected high risk community-dwelling individuals “By Nadarajah, et al

A. Summary:

The investigators used the OPTIMISE-1 randomised controlled trial (RCT) aiming to test the effect of community-based specialist-led identification and management of cardio-renal-metabolic-pulmonary (CRMP) disease and risk factors in comparison to usual care on use of therapeutic interventions over a follow up of six months among high FIND-AF risk community dwelling individuals.

The authors assumed that the OPTIMISE-1 RCT may provide novel data for a specialist-led intervention in primary care in a high risk cohort identified by a ML algorithm to improve the treatment of CRMP diseases

Strengths:

1. **Clinical Relevance**: Integrating an ML model into a randomized clinical trial aimed at early intervention in high-risk populations is commendable and very much in line with current priorities in healthcare innovation and population health.

2. **Use of a Certified Tool**: FIND-AF's certification as a UKCA Class I medical device supports the safety and reliability of its deployment in clinical workflows. The trial's foundation on a validated tool lends credibility to the approach.

3. **Well-Designed Trial Framework**: The trial benefits from a clear stratification strategy, a pragmatic design (PROBE), and integration within existing NHS infrastructure, which improves feasibility and generalizability.

Areas for Improvement:

1. **Lack of Technical Detail on the ML Model**: The protocol does not specify what type of model was used (e.g., gradient boosting, neural network), nor does it provide detail on the features selected, data preprocessing, or how the model handles missing values. A concise technical summary or reference would enhance transparency.

2. **Generalizability and Bias Assessment**: There is no discussion of whether the algorithm was tested across different demographics (e.g., age, sex, ethnicity). Since ML-driven interventions can exacerbate healthcare inequities, it would be important to include a fairness audit or subgroup analysis plan.

3. **Repurposing Beyond AF**: While it is logical to assume that those at high risk of atrial fibrillation may also be at higher risk of other CRMP conditions, this repurposing of the algorithm should be supported by validation results for those additional outcomes. Presenting evidence of predictive power beyond AF would solidify this assumption.

4. **Interpretability and Clinical Use**: The manuscript does not explain how the FIND-AF score is communicated to clinicians or patients. For the intervention to be truly usable, clinicians need interpretable outputs or contributing factors, rather than a black-box score alone.

5. **No Performance Metrics Shared**: The protocol does not report any evaluation metrics such as AUC, sensitivity, specificity, or calibration plots. At a minimum, these should be summarized from the cited validation study.

6. **Model Drift and Monitoring**: There is no mention of how performance degradation over time will be monitored, especially as healthcare data evolves. A plan to evaluate and potentially recalibrate the model would be reassuring.

Conclusion:

This is an exciting and forward-thinking clinical protocol that aligns well with the direction of personalized and AI-driven care. However, the manuscript would benefit from greater transparency around the ML algorithm's design, validation, fairness, and clinical usability. Strengthening these areas will enhance both the methodological rigor and practical relevance of the study.

I support publication of this protocol pending the authors' consideration and clarification of the above points.

Major Compulsory Revisions

• See above

Level of interest: N/A

Quality of written English: should be revised

Statistical review: not needed

Declaration of competing interests: 'I declare that I have no competing interests'

VERSION 1 - AUTHOR RESPONSE

Reviewers Comments
Strengths:

1. **Clinical Relevance**: Integrating an ML model into a randomized clinical trial aimed at early intervention in high-risk populations is commendable and very much in line with current priorities in healthcare innovation and population health.

Author reply
We thank the reviewer for the kind comment

2. **Use of a Certified Tool**: FIND-AF's certification as a UKCA Class I medical device supports the safety and reliability of its deployment in clinical workflows. The trial's foundation on a validated tool lends credibility to the approach.

Author reply
We thank the reviewer for the kind comment

3. **Well-Designed Trial Framework**: The trial benefits from a clear stratification strategy, a pragmatic design (PROBE), and integration within existing NHS infrastructure, which improves feasibility and generalizability.

Author reply
We thank the reviewer for the kind comment
Areas for Improvement:

1. **Lack of Technical Detail on the ML Model**: The protocol does not specify what type of model was used (e.g., gradient boosting, neural network), nor does it provide detail on the features selected, data preprocessing, or how the model handles missing values. A concise technical summary or reference would enhance transparency.

Author reply
We have now added more technical details about the FIND-AF algorithm. Given the development and validation of FIND-AF has been published previously, we hope the Reviewer will accept us describing it in brief in this manuscript.

Manuscript change
Derivation and validation of the FIND-AF score has been described elsewhere. [10] Briefly, it is a random forest classifier trained to predict AF within a 6 month prediction horizon. Random forest is a machine learning method consisting of many individual decision trees that operate as an ensemble. FIND-AF was trained using 10-fold cross validation, and included variables were restricted to age, sex, comorbidities and ethnicity to enhance scalability and international generalisability. [10] The predictor variables were selected from a prior systematic review. [11] Ethnicity information is routinely collected in the UK NHS and FIND-AF also included an 'ethnicity unrecorded' category where it was unavailable because missingness was considered to be informative. [10] Thus none of the variables are expected to be missing in routine data, and the algorithm could be applied at scale across a dataset of over 2 million UK primary care EHRs in the original derivation paper. [10] The performance of FIND-AF for prediction of incident AF was found to be equally robust and good-excellent in men and women and across ethnic groups. [10] The importance of variables for calculation of the FIND-AF score is demonstrated by the Gini impurity measure. [10]

2. **Generalizability and Bias Assessment**: There is no discussion of whether the algorithm was tested across different demographics (e.g., age, sex, ethnicity). Since ML-driven interventions can exacerbate healthcare inequities, it would be important to include a fairness audit or subgroup analysis plan.

Author reply
We completely agree with the Reviewer that generalisability and bias assessment are important when considering the application of machine learning data-driven approaches. The CPRD dataset that the algorithm was trained in representative of the UK population in terms of age, sex and ethnicity. Furthermore the original development and validation paper demonstrated that FIND-AF prediction performance was robust and good to excellent in men and women, and across ethnic groups. We also found the association of high FIND-AF risk and incident cardio-renal-metabolic-pulmonary disease was in both men and women and in both younger and older participants. We now describe this in the manuscript.

Manuscript change
The performance of FIND-AF for prediction of incident AF was found to be equally robust and good-excellent in men and women and across ethnic groups. [10]

On subgroup analysis, higher FIND-AF risk, compared with lower FIND-AF risk, was associated with increased incidence for each of the outcomes in both men and women and in younger (age 30-64 years) and older (age ≥65 years) patients. [8]

3. **Repurposing Beyond AF**: While it is logical to assume that those at high risk of atrial fibrillation may also be at higher risk of other CRMP conditions, this repurposing of the algorithm should be supported by validation results for those additional outcomes. Presenting evidence of

predictive power beyond AF would solidify this assumption.

Author reply
We have now added a section reporting the evidence for how high FIND-AF is associated with incident cardio-renal-metabolic-pulmonary disease and cardiovascular death.

Manuscript change
FIND-AF for incident cardio-renal-metabolic-pulmonary diseases
In a follow-on analysis using routinely collected UK primary care EHRs, we found higher FIND-AF risk, compared with lower FIND-AF risk, was associated with incident chronic obstructive pulmonary disease (cumulative incidence per 1000 persons at 10 years 395.8; hazard ratio 2.02, 95% CI 2.00-2.05; median time to event 2.68 years), chronic kidney disease (245.2; 6.85, 6.70-7.00; 5.44), heart failure (124.7; 12.54, 12.08-13.01; 4.06), diabetes mellitus (123.3; 2.05, 2.00-2.10; 3.45), stroke/transient ischaemic attack (118.9; 8.07, 7.80-8.34; 4.27), myocardial infarction (69.6; 5.02, 4.82-5.22; 4.32), peripheral vascular disease (44.6; 6.62, 6.28-6.98; 4.28), valvular heart disease (37.8; 6.49, 6.14-6.85; 4.54), aortic stenosis (18.7; 9.98, 9.16-10.87; 4.41), and death from any cause (273.9; 10.45, 10.23-10.68; 4.75). The higher risk group constituted 74% of deaths from cardiovascular or cerebrovascular causes (8 582/11 676). On subgroup analysis, higher FIND-AF risk, compared with lower FIND-AF risk, was associated with increased incidence for each of the outcomes in both men and women and in younger (age 30-64 years) and older (age ≥65 years) patients.

4. **Interpretability and Clinical Use**: The manuscript does not explain how the FIND-AF score is communicated to clinicians or patients. For the intervention to be truly usable, clinicians need interpretable outputs or contributing factors, rather than a black-box score alone.

Author reply
We agree with the Reviewer that usability is important. We have now added a section for this in the in the limitations section. We also agree that explainability is important. We have added a section describing how gini impurity measure shows the most important variables for prediction and was described in the original model development paper (doi: 10.1136/heartjnl-2022-322076)

Manuscript change
The importance of variables for calculation of the FIND-AF score is demonstrated by the Gini impurity measure. [10]

Limitations
Fourth, for translation to clinical practice the optimum approach to providing the FIND-AF risk stratification to primary care teams needs to be defined. Formation of a FIND-AF digital health platform that can integrate with primary care EHRs and provide contemporaneous estimates of FIND-AF risk at the patient level is the subject of funding applications. A qualitative study has already demonstrated that primary care healthcare professionals are enthusiastic to be able to utilise risk stratification through FIND-AF, and recommended the formation of high FIND-AF risk lists at the practice level as a medium through which the practice could organise clinical activities.

5. **No Performance Metrics Shared**: The protocol does not report any evaluation metrics such as AUC, sensitivity, specificity, or calibration plots. At a minimum, these should be summarized from the cited validation study.

Author reply
We have now added performance metrics from the original development and validation study for FIND-AF.

Manuscript change

FIND AF Score
Model performance for FIND-AF was determined using a holdout test dataset with internal bootstrap validation with 200 samples. The area under recriever operating characteristic curve (AUC) for incident AF was 0.824 (95% CI 0.814-0.834).[10]

FIND-AF for incident cardio-renal-metabolic-pulmonary diseases
In a follow-on analysis using routinely collected UK primary care EHRs, we found higher FIND-AF risk, compared with lower FIND-AF risk, was associated with incident chronic obstructive pulmonary disease (cumulative incidence per 1000 persons at 10 years 395.8; hazard ratio 2.02, 95% CI 2.00-2.05; median time to event 2.68 years), chronic kidney disease (245.2; 6.85, 6.70-7.00; 5.44), heart failure (124.7; 12.54, 12.08-13.01; 4.06), diabetes mellitus (123.3; 2.05, 2.00-2.10; 3.45), stroke/transient ischaemic attack (118.9; 8.07, 7.80-8.34; 4.27), myocardial infarction (69.6; 5.02, 4.82-5.22; 4.32), peripheral vascular disease (44.6; 6.62, 6.28-6.98; 4.28), valvular heart disease (37.8; 6.49, 6.14-6.85; 4.54), aortic stenosis (18.7; 9.98, 9.16-10.87; 4.41), and death from any cause (273.9; 10.45, 10.23-10.68; 4.75). The higher risk group constituted 74% of deaths from cardiovascular or cerebrovascular causes (8 582/11 676). On subgroup analysis, higher FIND-AF risk, compared with lower FIND-AF risk, was associated with increased incidence for each of the outcomes in both men and women and in younger (age 30-64 years) and older (age ≥65 years) patients.

6. **Model Drift and Monitoring**: There is no mention of how performance degradation over time will be monitored, especially as healthcare data evolves. A plan to evaluate and potentially recalibrate the model would be reassuring.

Author reply
We completely agree with the reviewer that we should have discussed this in the protocol. We have discussed this in the original protocol for the development of the FIND-AF score (doi: x). We have now added this in the limitations section.

Limitations
Fifth, model performance and association of model score with outcomes may change over time as the prevalence of predictors and association of predictors with the outcomes changes over time. As discussed in the original protocol for the development and validation of FIND-AF, the model itself could be reviewed and updated by a pre-specified expert consensus group on an annual basis after incorporating evidence from post-service utilization and the curation of more data.

VERSION 2 - REVIEW

**Reviewer**	**1**
**Name**	**Ibrahim, Mahmoud Ashraf**
**Affiliation**	**EDC , Center for Diabetes Education, Diabetes**
**Date**	**07-Jul-2025**
**COI**	

thanks, I have no further comments
